# Clinical predictors of suicide reattempts in the VigilanS follow-up program

**DOI:** 10.1192/j.eurpsy.2026.10213

**Published:** 2026-07-31

**Authors:** A. Marie, S. Berrouiguet, G. Vaiva, M. Walter, P. Courtet, G. Quellec

**Affiliations:** 1 Inserm; 2 CHU Brest, Brest; 3 Psychiatry, CHU Lille, Lille; 4 Psychiatry, CHU Brest, Brest; 5 Department of Emergency Psychiatry and Acute Care, CHU Montpellier, Montpellier; 6 UMR 1101, LaTIM, Inserm, Brest, France

## Abstract

**Introduction:**

Suicide reattempt (SR) rates are high in the postdischarge period for a suicide attempt (SA). Brief contact interventions (BCIs) aim to prevent SR by recontacting patients after discharge through calls or messages. A nationwide BCI called VigilanS has been deployed in France regions since 2015.

**Objectives:**

The objective of this study was to establish the incidence of suicide reattempt according to levels of risk, urgency, and dangerousness determined during phone calls.

**Methods:**

After being discharged from the hospital following an SA, patients are invited to join the program which provides phone calls follow-up for 6 months with continued monitoring for an additional 6 months if suicide risk is high (using the Columbia Scale20) or in case of an SR during follow up.

We analyzed data from 937 patients (71.6% women, 28.4% men) who entered the program between 07/03/2019 and 13/09/2021, with follow-up information collected until 21/01/2025.

Time-to-event analyses were performed to assess the predictive value of clinicians’ ratings of risk, urgency, and dangerousness on suicide reattempts.

Kaplan-Meier survival curves were used to estimate the cumulative probability of remaining free from reattemps over time, and differences between groups were tested using the log-rank test. Furthermore, we applied Cox proportional hazards models, which estimate hazard ratios (HR) reflecting the relative risk of SR for patients rated as moderate or high compared to those rated as low.

**Results:**

Survival curves analyses showed that the risk score significantly differentiated patients in terms of reattemps (global log-rank p=0.028, and Low vs High p=0.009). Neither urgency nor dangerousness were predictive (p>0.4).

However, survival curves suggested that patients rated “High” in any of the three scores tended to show less favorable outcomes, which indicates an increased vulnerability at the highest levels.

In Cox regression (cf forest plot), the risk score showed the strongest trend toward association with SR (HR>1 for both Moderate vs Low, and High vs Low), although not statistically significant. Urgency and dangerousness scores were not associated with SR, as HR was closer to 1 for both.

**Image 1:**
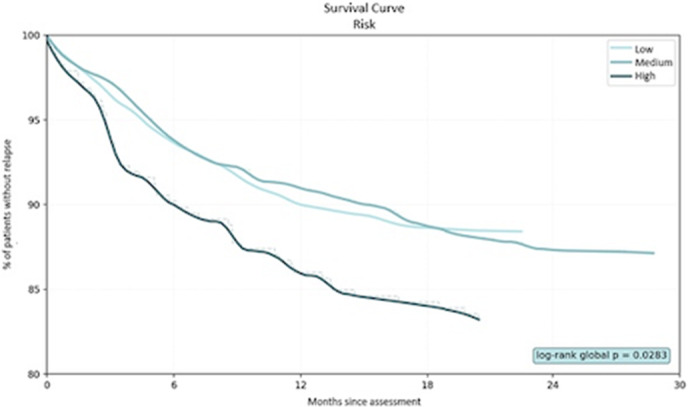
Image 1: Long description.

**Conclusions:**

Among the three clinical ratings assessed, only the risk score demonstrated partial predictive value for SR, while urgency and dangerousness did not. These findings suggest that predictive accuracy of current clinical assessments used in BCI can be limited. Future directions include developing AI-based approaches combining multiple data sources (electronic health records, phone call recordings, clinical notes) to improve risk assessment and to tailor BCI more effectively.

**Disclosure of Interest:**

None Declared

## Long descriptions

### Image 1: Long description

The line graph is titled Survival Curve Risk. The Y-axis represents the percentage of patients without relapse, ranging from 80 to 100 in increments of 5. The X-axis represents months since assessment, ranging from 0 to 30 in increments of 6.

Three distinct curves descend from the top-left corner at 100 percent:

* Low Risk (light blue line): Shows a gradual decline, ending at approximately 88 percent around month 22.

* Medium Risk (medium blue line): Follows a similar trajectory to the low risk group but extends further to month 29, ending at approximately 87 percent.

* High Risk (dark blue line): Shows the steepest decline with a wavy pattern, dropping to approximately 83 percent by month 20.

A legend in the top right identifies the three risk levels. In the bottom right corner, a text box states log-rank global p = 0.0283. Faint dashed lines follow the path of the solid curves, likely representing confidence intervals or raw data steps.

Navigate back to Image 1:.

